# Random Combinatorial Gradient Metasurface for Broadband, Wide-Angle and Polarization-Independent Diffusion Scattering

**DOI:** 10.1038/s41598-017-16910-4

**Published:** 2017-11-29

**Authors:** Yaqiang Zhuang, Guangming Wang, Jiangang Liang, Tong Cai, Xiao-Lan Tang, Tongfeng Guo, Qingfeng Zhang

**Affiliations:** 1 Air and Missile Defense College, Air Force Engineering University, Xi’an, 710051 China; 2 Southern University of Science and Technology, Shenzhen, 518055 China

## Abstract

This paper proposes an easy, efficient strategy for designing broadband, wide-angle and polarization-independent diffusion metasurface for radar cross section (RCS) reduction. A dual-resonance unit cell, composed of a cross wire and cross loop (CWCL), is employed to enhance the phase bandwidth covering the 2π range. Both oblique-gradient and horizontal-gradient phase supercells are designed for illustration. The numerical results agree well with the theoretical ones. To significantly reduce backward scattering, the random combinatorial gradient metasurface (RCGM) is subsequently constructed by collecting eight supercells with randomly distributed gradient directions. The proposed metasurface features an enhanced specular RCS reduction performance and less design complexity compared to other candidates. Both simulated and measured results show that the proposed RCGM can significantly suppress RCS and exhibits broadband, wide-angle and polarization independence features.

## Introduction

With the rapid development of detection and stealth technology, stealth military platform designs have attracted more and more attention. Stealth technology is mainly classified into two categories: shaping and absorbing incident waves^1–3^. The former category of technology redirects scattered waves away from the backward direction, whereas the second category transforms the incident waves into heat by loading radar absorber materials (RAM) on the scatters. However, both of these technologies suffer from design complexity and a limited operational band.

For the past several decades, metamaterials based on artificial structures have been proposed to achieve intriguing electromagnetic (EM) properties that cannot be obtained by using materials in nature^4–6^. Metamaterials are widely applied in stealth technologies, such as invisibility cloaks^7–9^ and perfect metamaterial absorbers (PMAs)^10,11^. The invisibility cloak provides an efficient way to smoothly bend incoming EM waves around the scatter, leading to an almost invisible object. However, their bulky structure, considerable thickness and extremely narrow bandwidth largely limit their applications. Meanwhile, PMAs, which are based on electric and magnetic resonances, suffer from a limited bandwidth as well. Therefore, it is highly desirable to develop a low-profile EM device to suppress backward scattering energy over a wide frequency band.

The metasurface is a two-dimensional (2D) equivalent of metamaterials, which allows manipulation of the propagation behavior of EM waves in a sub-wavelength scale along the propagation direction^12–15^. It can serve as an alternative approach to address the bandwidth and thickness issues of metamaterial-based stealth technology. Recently, many RCS reduction techniques using metasurfaces have been comprehensively demonstrated in both microwave and terahertz frequencies^16–35^. In ref.^16^, an artificial magnetic conductor (AMC) and a perfect electric conductor (PEC) arranged in a thin layer using a chessboard-like configuration were proposed to divide the scattering wave into four main lobes along the diagonal directions. Later, a combination of hexagonal and triangle AMC structures in a chessboard-like configuration was introduced to enhance the operational bandwidth and to further reduce the bistatic RCS^17,18^. A phase gradient metasurface (PGM) was also proposed to steer backscatter waves to predefined directions^19,20^. However, both chessboard-like metasurfaces and PGMs present a fixed scattering angle and relatively high bistatic RCS. To overcome these drawbacks, diffusion metasurfaces have been proposed to randomly diffuse the scattering waves into the upper half of space^21–35^. The diffusion metasurfaces were realized by arranging meta-particles in a size-variant or orientation-variant manner, resulting in a broadband and wide-angle RCS reduction with low-profile properties^21–28^. Although some diffusion metasurfaces were also designed with the aid of an optimization algorithm to achieve excellent low-scattering performances in microwave and terahertz frequencies^29–34^, the random distribution approach is much more efficient. It provides a good tradeoff between performance and design complexity. In ref.^35^,a new concept of coding phase gradient metasurface was proposed to manipulate the scattering waves with more flexibility, which was realized by designing an identical phase gradient into each coding element. However, diffusion was achieved by randomly coding the initial phase of the coding element, and the RCS reduction mechanism is similar to that of conventional diffusion metasurfaces. In ref.^36^,a dual-layer, anisotropic CWCL unit cell was used to design a bifunctional metasurface by orthogonal excitations.

To date, most of the diffusion metasurfaces have been realized by randomly arranging supercells, and each supercell consists of uniform unit cells^21–34^. However, the specular reflection of this type of diffusion metasurface cannot be dissolved. In this paper, a novel diffusion strategy is proposed for diffuse scattering in various directions and away from the specular reflection direction. The strategy was realized using a combination of supercells possessing different phase gradient directions instead of different phases and features an easy design and efficient manipulation of the scattering waves. Eight types of supercells were designed to deflect the scattering into eight different directions with a step of π/4 across the azimuth plane. Randomly arranging these supercells enables one to completely disturb the phase coherences of the different unit cells and to facilitate the diffusion scatterings. The designed diffusion metasurface features a broadband, wide-angle and polarization-independent performance in RCS reduction.

## Results

### Design Principle

The array antenna theory is used to illustrate the working mechanism of the random combinatorial gradient metasurface. Considering a metasurface composed of *M* × *N* elements under a normal plane wave incidence, the scattering patterns of the entire metasurface can be expressed by superposing the contributions of all the elements, as shown in Eq. (1).

1 $${\vec{E}}_{total}=\sum _{m}\sum _{n}{\vec{E}}_{m,n}\exp [j{k}_{0}(m{d}_{x}\,\sin \,\theta \,\cos \,\phi +n{d}_{y}\,\sin \,\theta \,\sin \,\phi )]$$

where ${\vec{E}}_{m,n}$ is the electric far-field pattern of the element (*m*, *n*); *θ* and *φ* are the elevation and azimuth angles, respectively; *d*_*x*_ and *d*_*y*_ denote the element period along the *x* and *y* directions, respectively; and *k*_0_ is the wave number in free space. Due to the all-reflection property of the elements, we can assume that these elements have identical scattering patterns and uniform reflection magnitudes but different reflection phases. Therefore, the electric field of each element is

2 $${\vec{E}}_{m,n}=\exp (j{\phi }_{mn}){\vec{E}}_{1}$$

**Figure 1:**
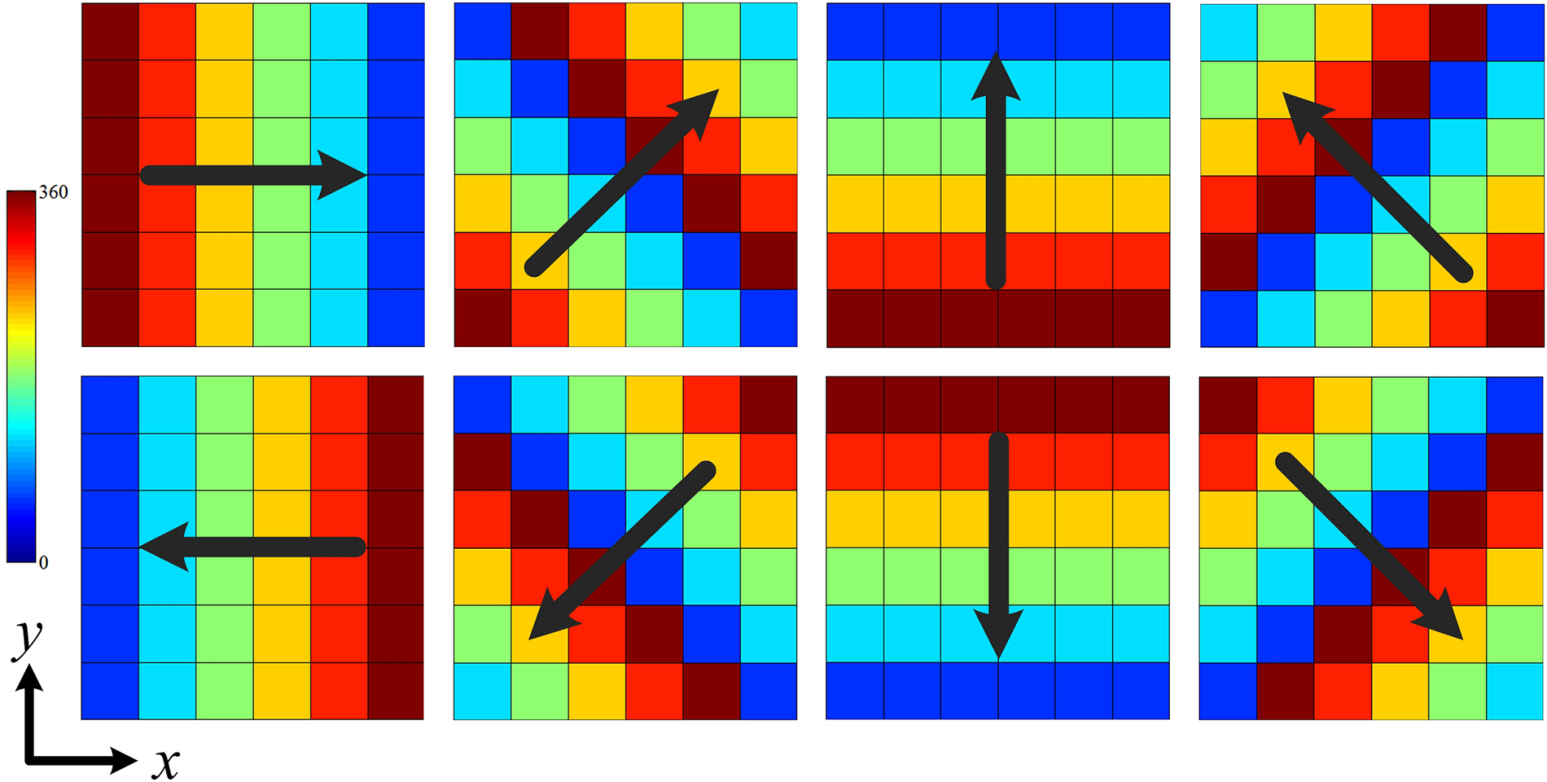
Phase distributions of the eight types of phase gradient elements.

Upon substitution into Eq. (1), this leads to

3 $${\vec{E}}_{total}={\vec{E}}_{1}\sum _{m}\sum _{n}\exp [j({\phi }_{mn}+{k}_{0}m{d}_{x}\,\sin \,\theta \,\cos \,\phi +{k}_{0}n{d}_{y}\,\sin \,\theta \,\sin \,\phi )]={\vec{E}}_{1}\cdot AF$$

where ${\vec{E}}_{1}$ is the electric far-field pattern of the basic element; and AF is the array factor. Since each element in a conventional random-diffusion metasurface is composed of several homogeneous unit cells, the scattering pattern of the entire metasurface is only determined by the configuration of the element. To manipulate the scattering pattern more flexibly, an additional gradient phase is added into the element. The phase gradient metasurface can redirect the reflection waves to a desired direction with respect to the phase gradient. Here, we design eight types of phase gradient elements (including horizontal, vertical and oblique directions in the phase gradient) across the azimuth plane. As shown in Fig. 1, each element is composed of 6 × 6 unit cells, and the phase gradient direction is rotated counterclockwise with a step of 45°.

**Figure 2:**
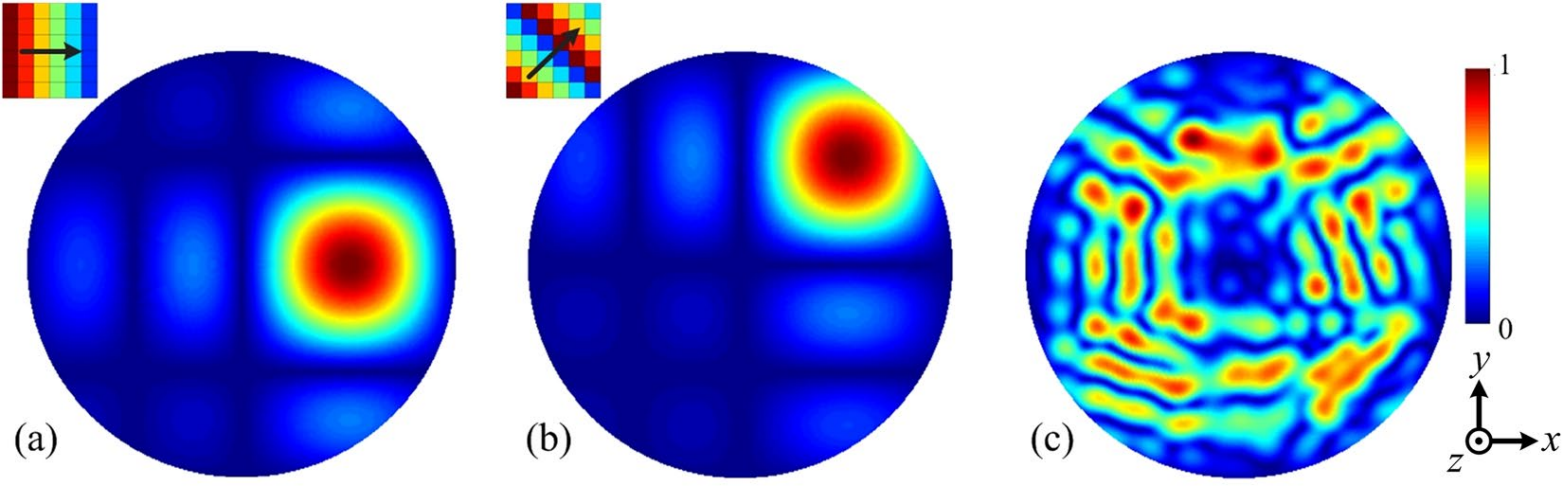
Theoretical scattering patterns of the (**a**) horizontal-gradient element, (**b**) oblique-gradient element, and (**c**) random combinatorial gradient metasurface.

To demonstrate the anomalous reflections of the proposed elements, we calculated the scattering patterns of the horizontal-gradient, oblique-gradient, and random combinatorial gradient metasurface under a normal incidence, as shown in Fig. 2. All the scattering patterns were normalized to their maximum intensity. Note in Fig. 2(a) and (b) that the reflected beams are reflected into different directions for both the horizontal-gradient and oblique-gradient cases. Therefore, one expects that a metasurface constructed by randomly distributing these eight types of elements can disperse scattering waves into various directions with very low backward scattering. Figure 2(c) shows the theoretical scattering pattern of a random combinatorial gradient metasurface consisting of 4 × 4 elements, which is consistent with our expectation.

### Unit Cell Design

**Figure 3:**
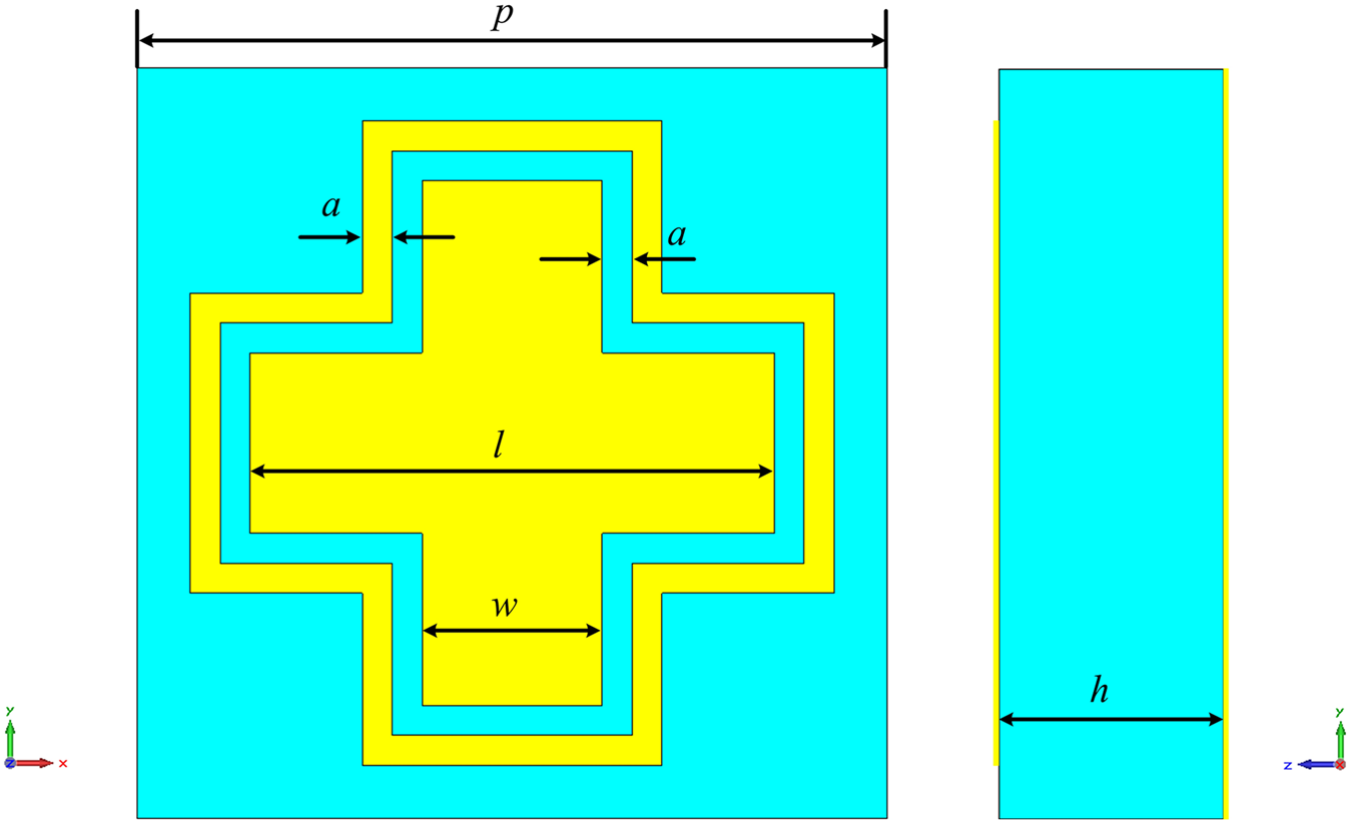
The front view (left) and side view (right) of the unit cell.

**Figure 4:**
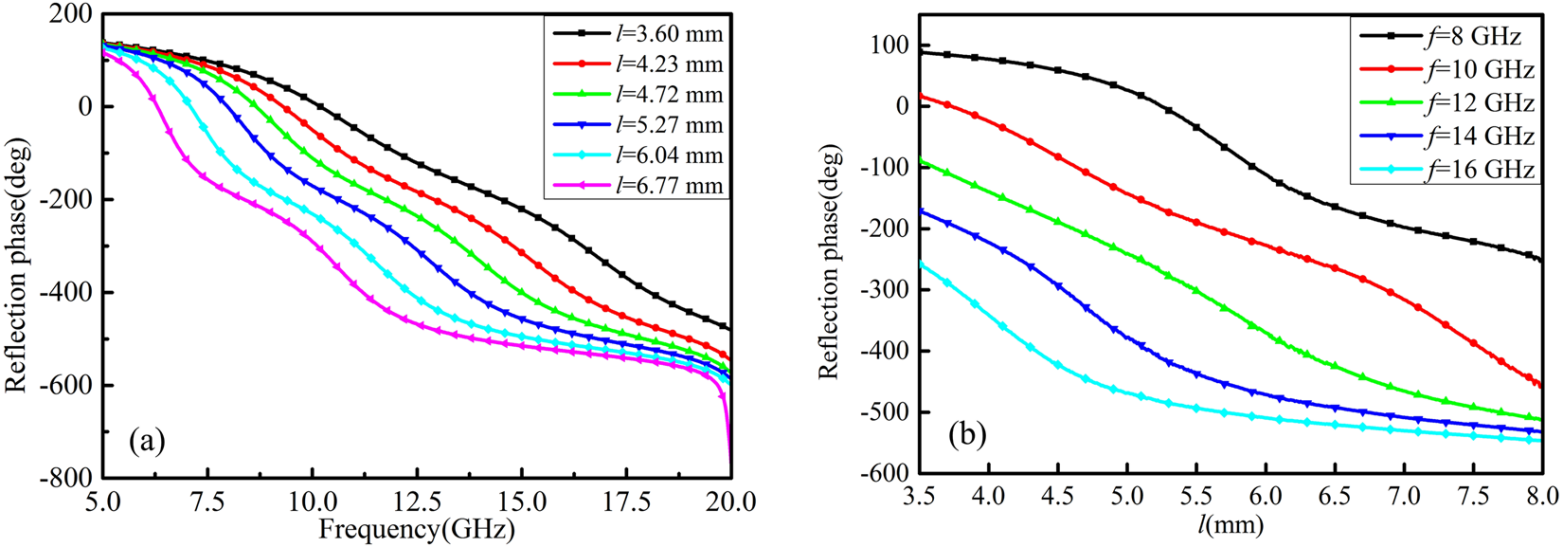
The reflection phases (**a**) for different values of *l* versus the frequency and (**b**) at different frequencies versus *l*.

To enhance the operational bandwidth, an isotropic, dual-resonance unit cell is employed as the basic reflective phasing element^36^, as shown in Fig. 3. The unit cell is a sandwich-like structure with a metallic structure on the top layer, an F4B substrate (with a thickness, *h*, of 3 mm and a dielectric constant of 2.65) in the middle, and a metallic ground plane as the bottom layer. The metallic structure is composed of a cross wire and cross loop, leading to two resonance frequencies close to each other. Furthermore, the proposed unit cell is an almost isotropic structure, which makes it insensitive to the polarization of the incident wave. An enhanced operational bandwidth and improved reflection phase range can be achieved. To efficiently manipulate the reflection phase, the optimum dimensions of the unit cell are chosen as *p* = 10 mm, *a* = 0.4 mm, and *w* = 2.4 mm. The simulated reflection phases are plotted in Fig. 4, which shows that the reflection phase can be manipulated with a variation of *l*. It is clearly seen in Fig. 4(a) that the curves are quite linear, and they are almost parallel to the variation of *l*, leading to an excellent broadband response. As shown in Fig. 4(b), the reflection phase curves at different frequencies with a variation of *l* are almost parallel as well, which is important to ensure a wide operational bandwidth.

### Phase Gradient Supercell Element

As a special type of metasurface, PGM has been widely used to achieve anomalous reflection performance. By delicately designing the phase gradient on PGM, the reflected wave can be redirected in the desired direction. As illustrated in Fig. 1, eight types of supercells must be implemented using the proposed unit cell in Fig. 3. As an example, the design approach for the oblique-gradient phase and horizontal-gradient phase supercell is shown. Other types can be designed in a similar manner.

**Figure 5:**
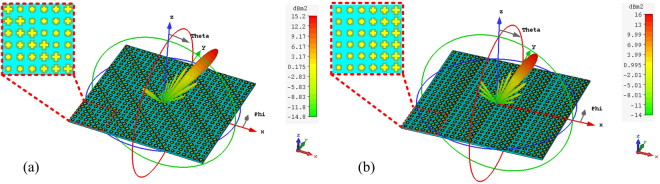
The 3D far-field scattering pattern for the (**a**) oblique-gradient case and (**b**) horizontal-gradient case at 10 GHz.

In the oblique-gradient case, the phase gradient can be projected in both the *x* and *y* direction, leading to an out-of-plane anomalous reflection. The reflected wave can be characterized by two angles, *θ*_*r*_ and *φ*_*r*_, which represent the reflected elevation angle and azimuth angle, respectively. As depicted in Fig. 5, we employ six unit cells with identical phase gradients along the *x* and *y* directions, i.e., $d{\rm{\Phi }}/dx=d{\rm{\Phi }}/dy$. In the case of the oblique-gradient phase, the generalized Snell’s law is illustrated as:

4 $$\sin \,{\theta }_{r}-\,\sin \,{\theta }_{i}=\frac{{\lambda }_{0}}{2\pi {n}_{i}}\sqrt{{(\frac{d{\rm{\Phi }}}{dx})}^{{\rm{2}}}+{(\frac{d{\rm{\Phi }}}{dy})}^{{\rm{2}}}}$$

where *θ*_*r*_ and *θ*_*i*_ are the reflection elevation angle and incidence angle, respectively; *λ*_0_ is the wavelength in free space; *n*_*i*_ represents the refractive index of the incidence medium; and $d{\rm{\Phi }}/dx$ ($d{\rm{\Phi }}/dy$) denotes the phase gradient along the *x* (*y*) direction. To realize a broadband anomalous reflection performance and to efficiently suppress backward RCS, we employ six unit cells to realize a phase gradient covering a 2π range to guarantee a preferable performance. Upon substitution of $d{\rm{\Phi }}/dx=d{\rm{\Phi }}/dy$, Eq. (4) is reduced to

5 $$\sin \,{\theta }_{r}-\,\sin \,{\theta }_{i}=\frac{\sqrt{2}{\lambda }_{0}}{2\pi {n}_{i}}\frac{d{\rm{\Phi }}}{dx}$$

The phase shift across a single unit cell is ${\rm{\Delta }}{\rm{\Phi }}=2\pi /n$, where *n* is the number of unit cells. Therefore, the phase gradient is computed as $d{\rm{\Phi }}/dx={\rm{\Delta }}{\rm{\Phi }}/p=2\pi /np$. Under normal incidence, *θ*_*r*_ is computed as ${\theta }_{r}=\arcsin (\sqrt{2}{\lambda }_{0}/np)$. Since the azimuth angle, *φ*_*r*_, satisfies the condition $\tan \,{\phi }_{r}=(d{\rm{\Phi }}/dy)/(d{\rm{\Phi }}/dx)$ = 1, it is fixed at 45°. In other words, the reflected waves are redirected in the diagonal plane. The oblique-gradient phase can be reduced to the horizontal-gradient phase when $d{\rm{\Phi }}/dy=0$. Therefore, the reflection angle in the horizontal-gradient case is calculated as ${\theta }_{r}=\arcsin ({\lambda }_{0}/np)$ and *φ*_*r*_ = 0°.

**Figure 6:**
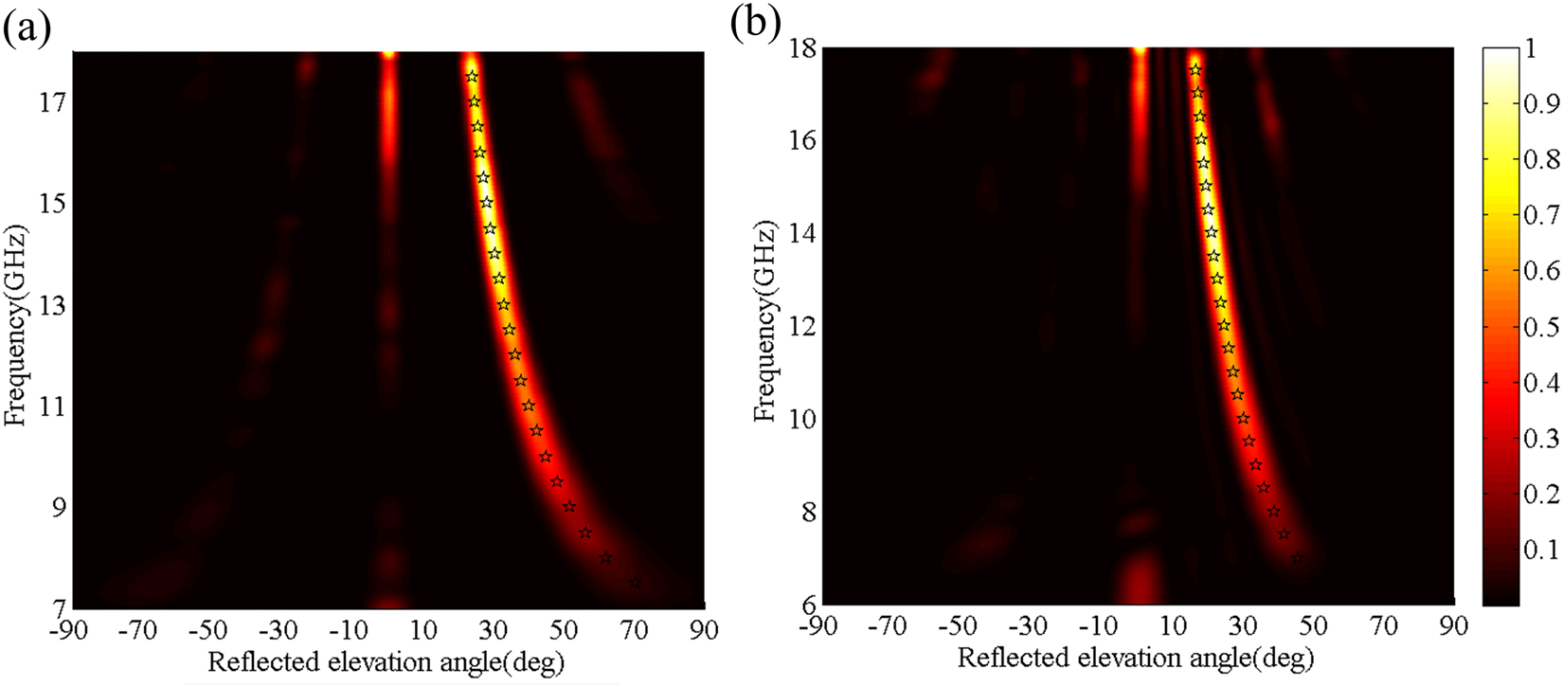
The normalized scattering intensity spectra of the simulated and theoretical results in (**a**) the diagonal plane for the oblique-gradient case and (**b**) *xoz* plane for the horizontal-gradient case under normal incidence. The star-shaped markers represent the traces of the theoretical maximum-intensity points.

**Figure 7:**
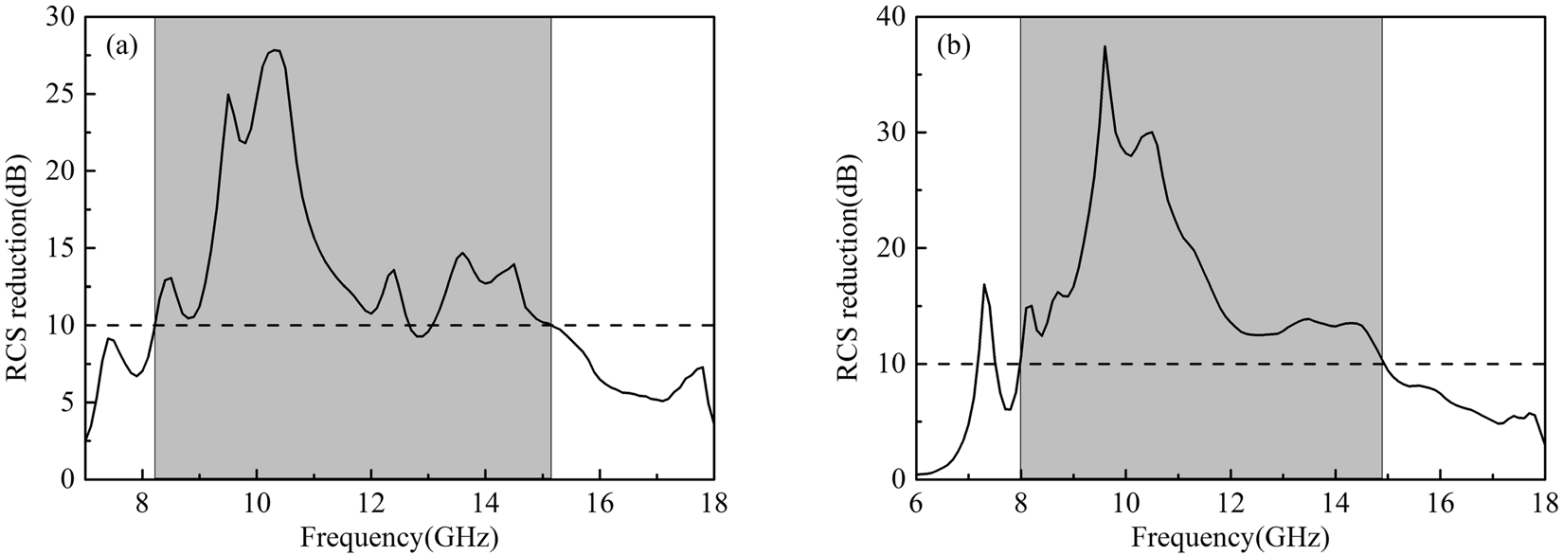
The RCS reduction spectra for the (**a**) oblique-gradient case and (**b**) horizontal-gradient case.

As an example, six unit cells with *l* = 3.6 mm, 4.23 mm, 4.72 mm, 5.27 mm, 6.04 mm and 6.77 mm are employed to design the oblique-gradient and horizontal-gradient supercells, as shown in Fig. 5. The phase-shift interval is ${\rm{\Delta }}{\rm{\Phi }}=60^\circ$ across each unit cell at 10 GHz. Both PGMs constructed by 4 × 4 supercells with 240 mm × 240 mm illuminated by a normal *x*-polarized plane wave were simulated in the CST Microwave Studio. The three-dimensional (3D) far-field scattering patterns are shown in Fig. 5. As expected, an out-of-plane anomalous reflection is realized for the oblique-gradient case, whereas the reflected waves are effectively redirected in a predefined direction in the *xoz* plane for the horizontal-gradient case. Furthermore, the normalized scattering intensity spectra are depicted in Fig. 6. The star-shaped marker in Fig. 6 denotes the theoretical value of the reflection angles, which was calculated by ${\theta }_{r}=\arcsin (\sqrt{2}{\lambda }_{0}/np)$ and ${\theta }_{r}=\arcsin ({\lambda }_{0}/np)$. The excellent agreement between the simulated and theoretical results indicates that the reflected energies are redirected in the predefined direction. In addition, this is the reason why the RCS can be reduced more than 10 dB within 8.2–15.1 GHz and 8.0–14.9 GHz for the oblique-gradient and horizontal-gradient, respectively, as shown in Fig. 7. One should note that this proposed scheme can arbitrarily manipulate the azimuth angle of the reflection by employing phase gradient supercells in different azimuth directions. However, the reflected elevation angles cannot be arbitrarily controlled.

### Random Combinatorial Gradient Metasurface

**Figure 8:**
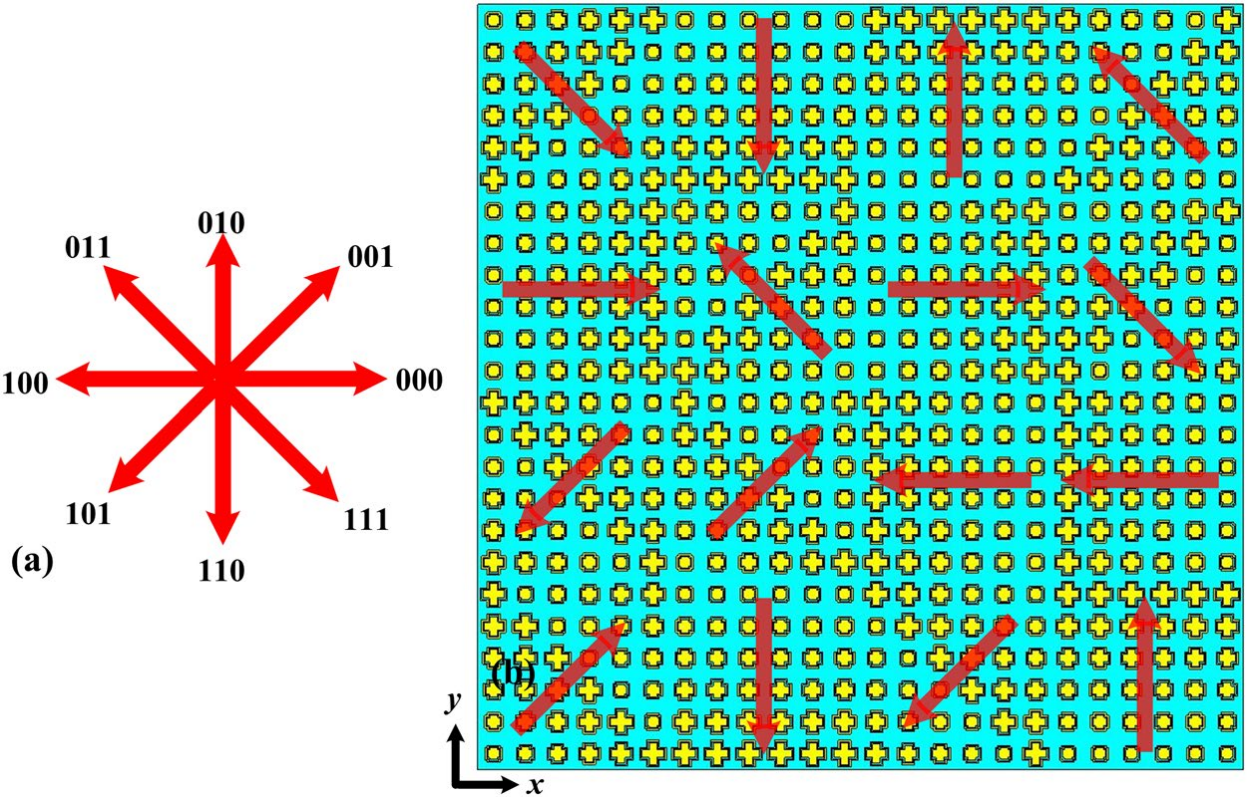
(**a**) Schematic of the 3-bit coding principle with eight types of phase gradient directions and (**b**) the layout of a certain RCGM (layout I).

As demonstrated above, the PGM can redirect the reflected waves to the desired direction with a high efficiency and broad operational band. To diffuse the reflection into various directions, one may randomly distribute different types of supercells to design the diffusion metasurface. Here, eight types of phase gradient supercells are employed across the azimuth plane. Therefore, a 3-bit coding method was adopted, and each type of phase gradient supercell was defined as a coding element, as shown in Fig. 8(a). Although there are many possibilities to determine the random sequence, three certain layouts in a finite sheet of 240 mm × 240 mm were investigated without a loss of generality. The different layouts were designed according to three pseudorandom 3-bit coding sequences generated by MATLAB. The entire metasurface was composed of 16 elements. Figure 8(b) shows layout I for this approach.

**Figure 9:**
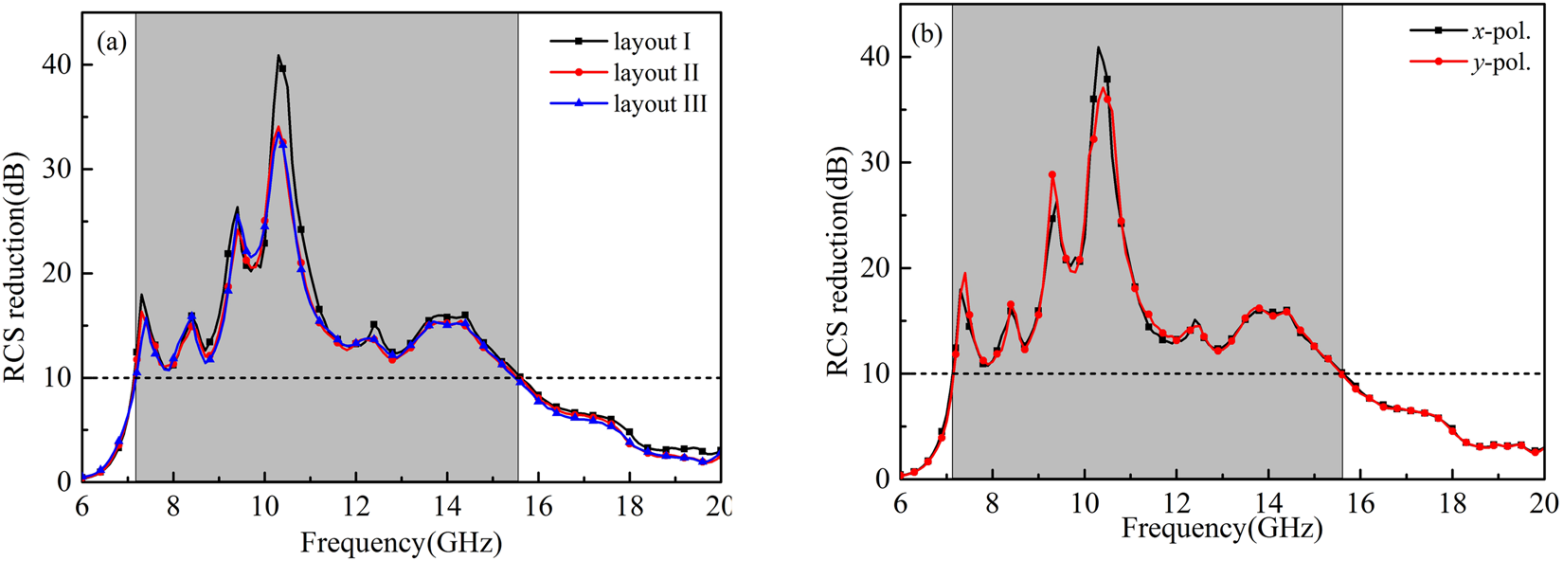
Monostatic RCS reduction of the (**a**) three layouts for *x*-polarized waves and (**b**) layout I for both *x*- and *y*-polarized waves under a normal incidence.

**Figure 10:**
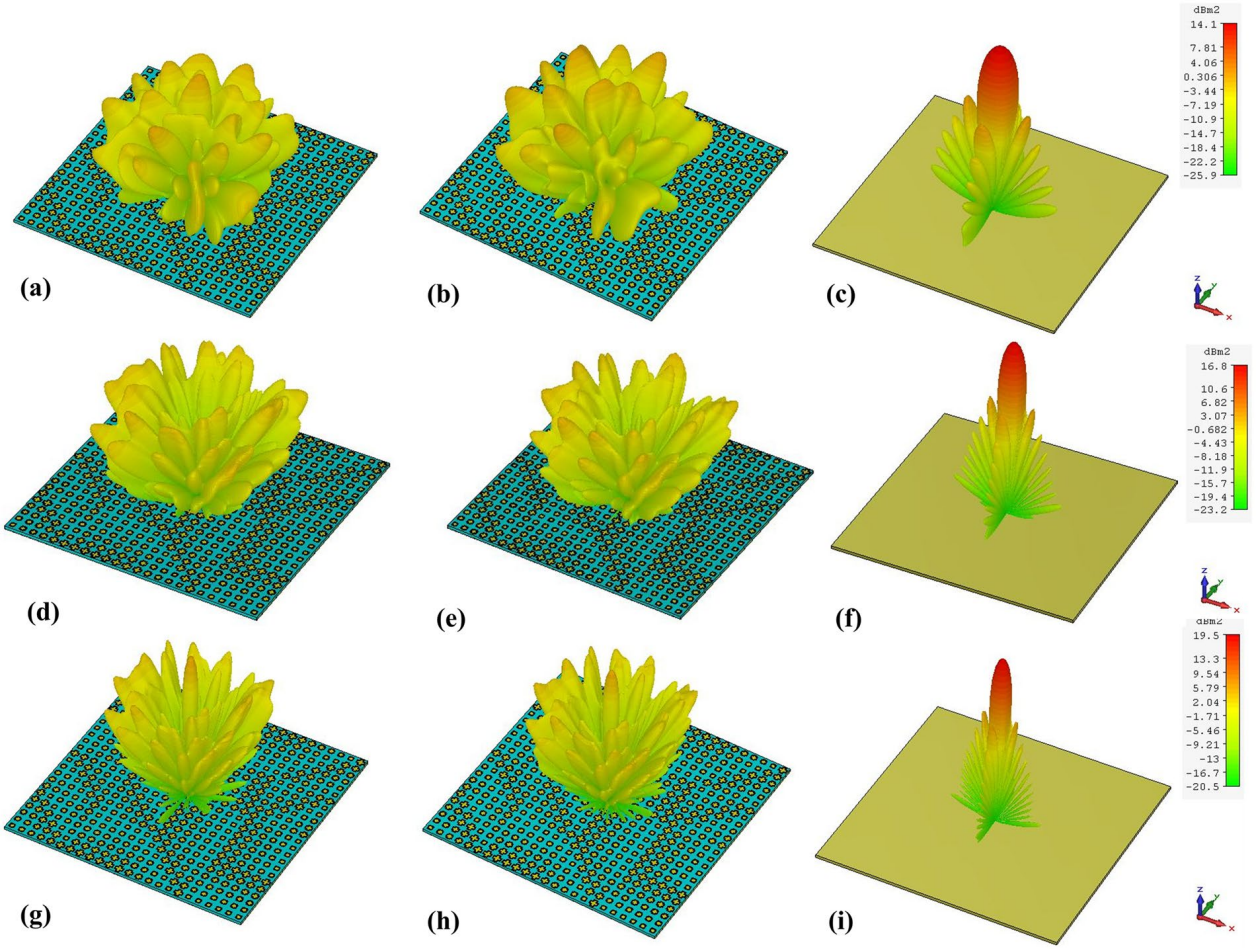
The 3D RCS patterns of RCGM under (**a**,**d**,**g**) *x*- and (**b**,**e**,**h**) *y*-polarized incident waves; (**c**,**f**,**h**) a metallic plate under *x*-polarized wave excitation at (**a–c**) 7.3 GHz, (**d–f**) 10.3 GHz and (**g–i**) 13.3 GHz.

Full-wave simulations of the three RCGM layouts were carried out in the CST Microwave Studio to validate the diffusion scattering performance. Figure 9(a) and (b) show the RCS reduction spectra of the three layouts and that of layout I in Fig. 8(b) under both *x*- and *y*-polarized plane wave normal incidences. The RCS reduction value is calculated using the reflection of a metallic plate with the same size as the reference. Note that all three RCGMs exhibit very similar curves and suppress the RCS by more than 10 dB within the frequency band from 7.1 to 15.6 GHz. For simplification, only layout I is investigated in the following section. As shown in Fig. 9(b), the proposed RCGM is insensitive to the polarizations, and the maximum RCS reduction value reaches 40 dB at 10.3 GHz. The slight frequency shift of 0.3 GHz is probably because the unit cell is designed using periodic boundary conditions for identical unit cells, whereas random unit cells are used in the final layout. Figure 10(a–c),(d–f) and (g–i) illustrate the simulated 3D far-field scattering patterns of RCGM under *x*- and *y*-polarized wave incidences and those of a metallic plate at 7.3, 10.3, and 13.3 GHz. It can be clearly seen that the diffusion behaviors of the scattered fields are observed in a wide frequency band, which contrasts with the case with a metallic plate. Furthermore, it is worth mentioning that no reflection occurs in the specular direction at 10.3 GHz, which corresponds to the minimum value in the RCS reduction spectra (see Fig. 9(b)). Also, the scattering patterns are almost the same for both the *x*- and *y*-polarized wave incidences. Thus, the proposed metasurface can suppress backward scattering for polarization-independent incident waves, which is very useful in practical applications.

**Figure 11:**
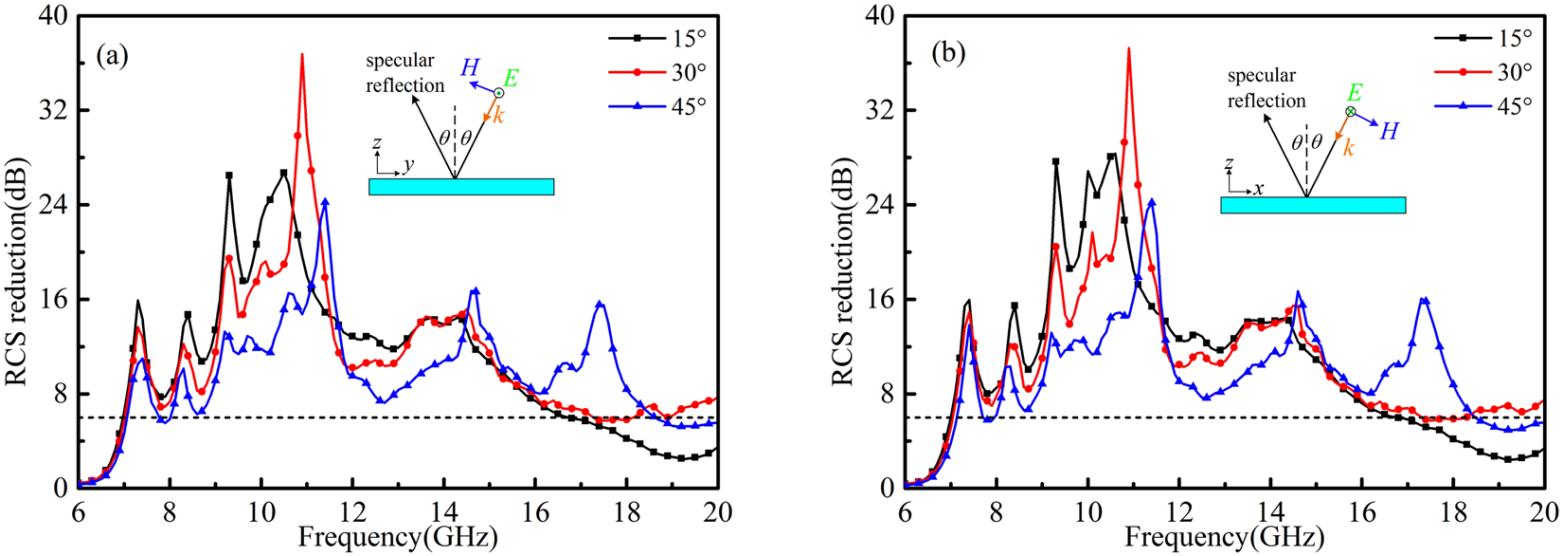
Monostatic RCS reduction under (**a**) *x*- and (**b**) *y*-polarized oblique incidences.

**Figure 12:**
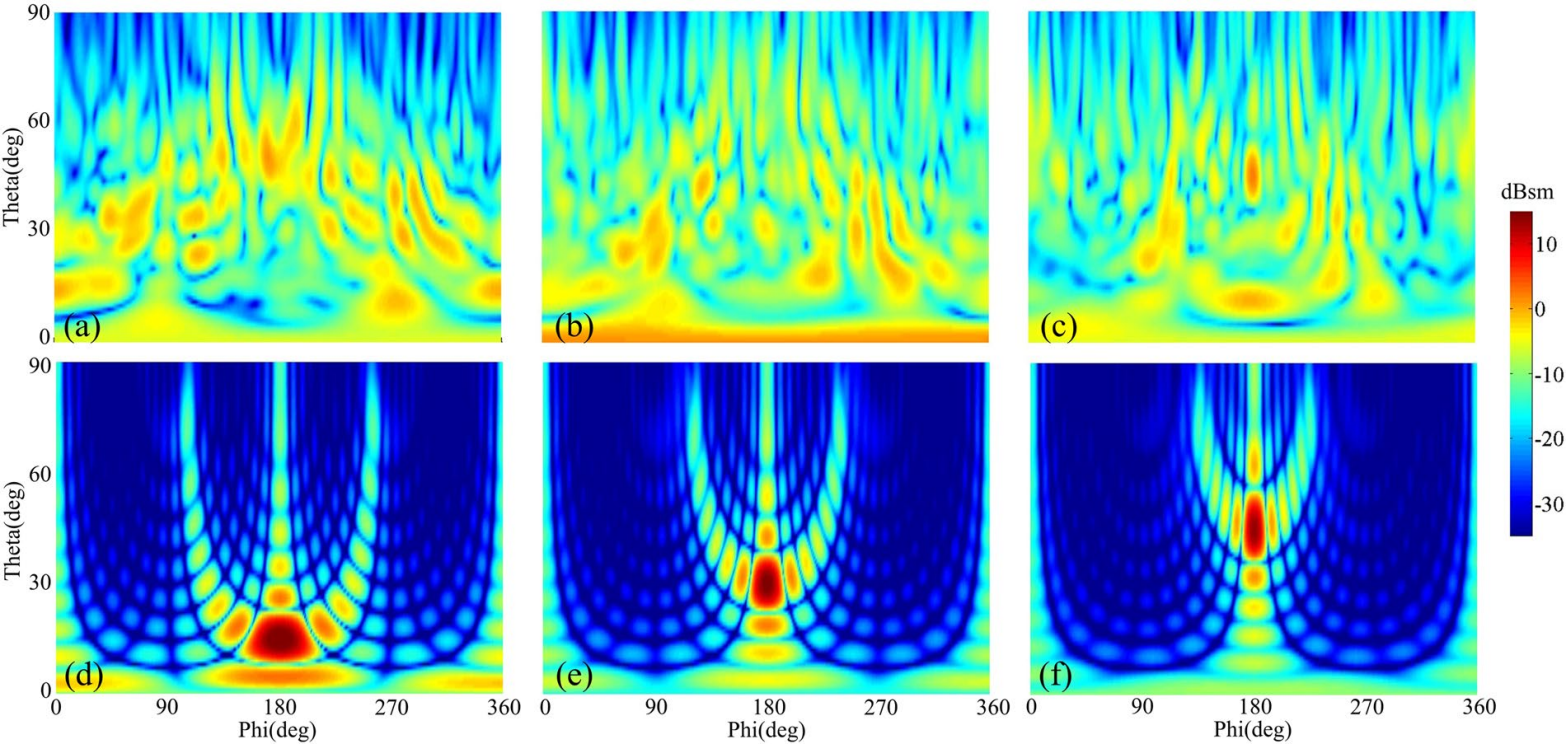
The scattering spectra of (**a**–**c**) RCGM and (**d**–**f**) a metallic plate under *y*-polarized wave incidences with different oblique incidence angles (15°, 30° and 45°) at 10.3 GHz.

To provide a comprehensive understanding of the angular performance of RCGM, the RCS reduction in the specular direction under oblique incidence is also investigated. Both *x*- and *y*- polarized waves from 15°, 30° and 45° are considered. As shown in Fig. 11, the RCS reduction performance gradually degraded due to phase aberrations with the increase in the incidence angle. However, a 6 dB RCS reduction can still be achieved in a broad frequency band which means less than 25% of the power is reflected back in the specular direction. Moreover, the peak exhibits a redshift as the incident angle increases, which is also attributed to the phase alteration. To demonstrate the diffusion performance of the RCGM under oblique incidence, the scattering spectra in the backward space of the RCGM under oblique incidence at 10.3 GHz are given in Fig. 12, and the spectra of a metallic plate is also afforded in the same color map scale for comparison. It can be clearly observed that most of the scattering energy was dispersed into numerous off-specular directions for the RCGM, which is totally different from the scattering distribution of the metallic plate. Therefore, both monostatic RCS and bistatic RCS were effectively suppressed.

### Fabrication and measurement result

**Figure 13:**
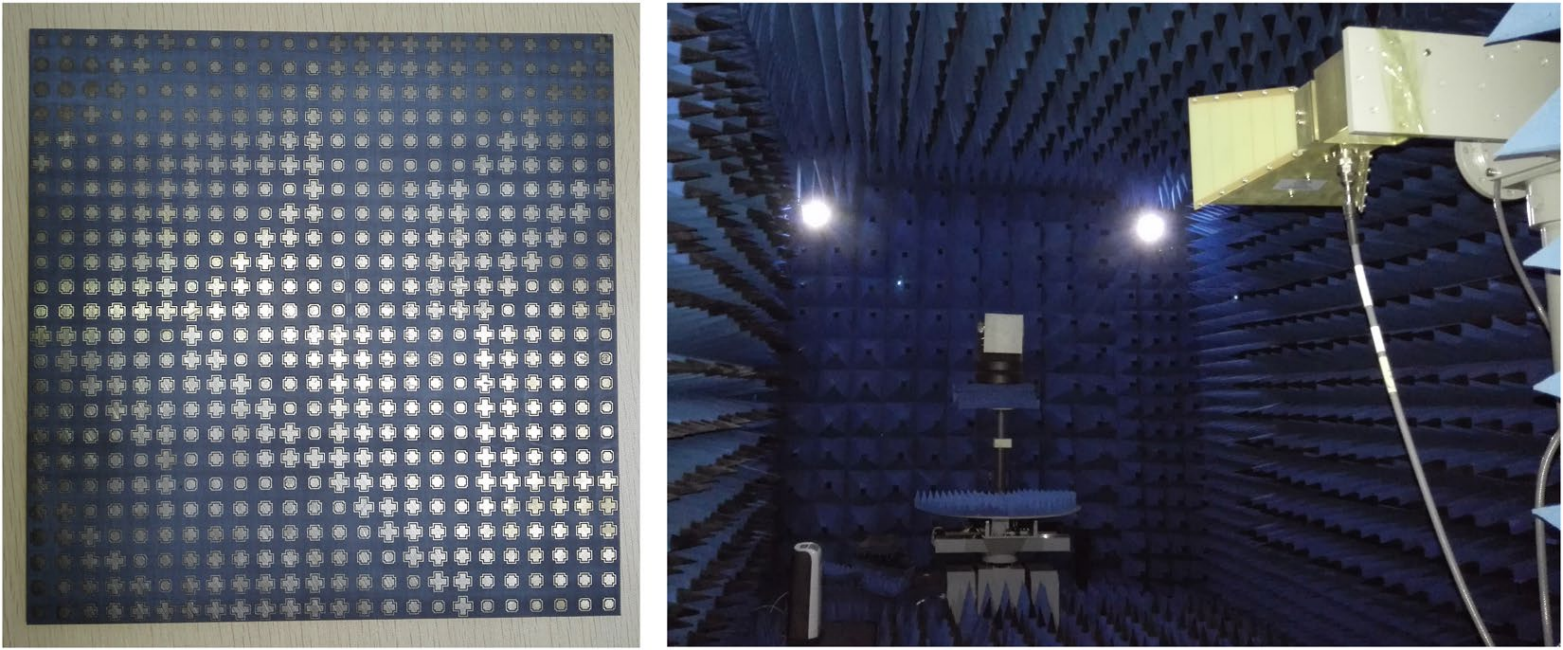
Photograph of the fabricated prototype (left) and the measurement setup(right).

**Figure 14:**
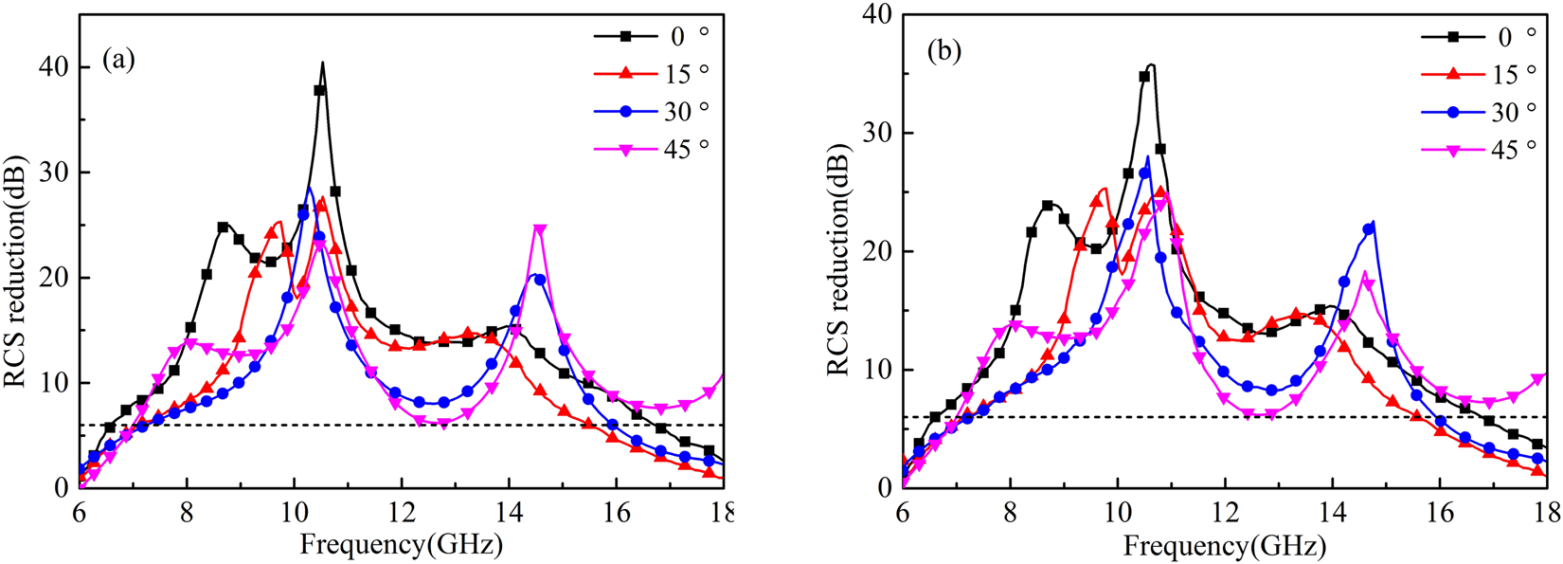
The measurement results of the RCS reduction for various incident angles; (**a**) *x*-polarized and (**b**) *y*-polarized waves.

To validate the performance of the proposed RCGM, a prototype was fabricated and measured. Figure 13 shows the photograph of the fabricated prototype and the experimental setup for the normal incidence. The inset in the upper-left of Fig. 13(b) shows the detailed setup of the transmitter and receiver. The prototype was manufactured by a stand print circuit board process technology. The upper metallic structure layer and bottom ground layer were 0.036 mm thick copper, and the middle dielectric substrate layer was an F4B board with a dielectric constant of 2.65 and a loss tangent of 0.001. The fabricated prototype, which has the same pattern as layout I in the simulation (Fig. 8(b)), has a total size of 240 mm × 240 mm. Two identical, wideband, ridged horn antennas operating from 1 to 18 GHz were used as the transmitter and receiver. The reflections of a purely metallic plate were also measured as a reference. Figure 14 shows the measured results of the RCS reduction spectra under *x*- and *y*-polarized waves impinging from 0°, 15°, 30° and 45° angles. Note that the RCS can be reduced by more than 6 dB within a wide frequency range under incidence angles less than 45°. Furthermore, the change tendency is consistent with the simulation results with the increment of the incident angle. Considering the fabrication and measurement errors, reasonable agreement can be found between the measured and simulated results.

## Discussion

In summary, a novel strategy to realize a diffusion metasurface was proposed for reducing the RCS with broadband, wide-angle and polarization-independent features. A prototype was constructed by randomly distributing eight types of PGM supercells on a metasurface. Each supercell was composed of six CWCL unit cells with a special phase gradient direction. The CWCL unit cell was employed to broaden the operational band covering the 2π phase range. Since the anomalous reflection angle of the regular PGM was determined by the phase gradient, the metasurface using different PGM supercells can scatter the incident waves to various directions in the backward half space. Both the simulated and measured results demonstrated that the designed metasurface effectively suppressed the backward RCS within the frequency range of 7.2–15.6 GHz. Furthermore, low reflection features were still maintained for wide-angle oblique incidences, various polarizations, and broadband operations. This work provides a new approach for suppressing the backward RCS.

## Methods

### Simulation

All the full-wave simulations were carried out by the commercial software, CST Microwave Studio. The frequency domain solver was utilized for the unit cell simulation. In addition, the unit cell boundary and open boundary were applied for both the *x* and *y* directions and the *z* direction, respectively. The full model of the metasurface was simulated by a time domain solver, and an open (add space) boundary was applied in all directions.

### Measurement

The reflection measurements were conducted in a microwave anechoic chamber. A pair of identical, wideband, ridged horn antennas was used as the transmitter and receiver, and the pair was connected to a vector network analyzer (AV 3672B). In the normal incidence case, the two antennas were placed in front of the prototype at a distance of 3 m to satisfy the far-field measurement conditions. The antennas were placed at the same height as the prototype in the measurement. To eliminate the interference of the environment, the function of the time-domain gating in the network analyzer was adopted in the experiments. The reflection performance was evaluated by the transmission parameter, *S*_21_, and the RCS reduction performance of the prototype was calibrated using a metallic plate that was the same size.
